# A PCB Electronic Components Detection Network Design Based on Effective Receptive Field Size and Anchor Size Matching

**DOI:** 10.1155/2021/6682710

**Published:** 2021-02-26

**Authors:** Jing Li, Weiye Li, Yingqian Chen, Jinan Gu

**Affiliations:** ^1^School of Mechanical Engineering, Jiangsu University, Zhenjiang 212000, China; ^2^School of Electronic Information and Electrical Engineering, Anyang Institute of Technology, Anyang 455000, China; ^3^School of Mechanical and Electrical Engineering, Guangdong University of Technology, Guangzhou 510006, China

## Abstract

Vision-based recognizing and positioning of electronic components on the PCB (printed circuit board) can improve the quality inspection efficiency of electronic products in the manufacturing process. With the improvement of the design and the production process, the electronic components on the PCB show the characteristics of small sizes and similar appearances, which brings challenges to visual object detection. This paper designs a real-time electronic component detection network through effective receptive field size and anchor size matching in YOLOv3. We make contributions in the following three aspects: (1) realizing the calculation and visualization of the effective receptive field size of the different depth layers of the CNN (convolutional neural network) based on gradient backpropagation; (2) proposing a modular YOLOv3 composition strategy that can be added and removed; and (3) designing a lightweight and efficient detection network by effective receptive field size and anchor size matching algorithm. Compared with the Faster-RCNN (regions with convolutional neural network) features, SSD (single-shot multibox detectors), and original YOLOv3, our method not only has the highest detection mAP (mean average precision) on the PCB electronic component dataset, which is 95.03%, the smallest parameter size of the memory, about 1/3 of the original YOLOv3 parameter amount, but also the second-best performance on FLOPs (floating point operations).

## 1. Introduction

As an essential component of electronic information products, electronic components must be assembled under the rules of the correct class and correct location in the manufacturing process of electronic products [1]. For a long time, the identification and positioning of electronic components on the PCB has become the technical key during manufacturing and assembling of electronic products. The application of machine vision technology for AOI (automatic optical inspection) can reduce production costs, improve inspection speed and inspection accuracy, and achieve 100% inspection [2–5]. Its efficiency and quality consistency are far superior to manual visual inspection. Especially in recent years, the convolutional neural network (CNN) has achieved great success in many computer vision fields, such as image classification, object detection [6–13], target tracking, target recognition, and semantic segmentation. More and more vision-based object detection systems have been widely used in the electronics manufacturing industry.

As we know, there are many kinds of electronic components and different shapes, and CNN simulates the visual cognition principle of the brain and retains the features of the object through dimensionality reduction, even if the object appears again when the scale, direction, and position are different to identify it. Therefore, it is necessary to combine the detection of electronic components with the CNN. Kuo et al. proposed a novel Graph Network block to refine the component features conditioned on each PCB. The mAP of electronic component detection on the testing PCBs can reach 65.3% [14]. Li et al. proposed an improved YOLOv3 algorithm that added one output layer sensitive to small targets and validated the algorithm effectiveness in a real PCB picture and virtual PCB picture test, including many PCB electronic components [15]. Huang et al. proposed a fast recognition method for electronic components in a stacked disordered scene. They used MobileNet instead of Darknet-53 in YOLOv3 to achieve lightweight and rapid speed [16]. From the above CNN-based electronic components detection framework, however, we found that those methods did not consider the different perspectives from the visual areas regarding the depths of network layers, bringing about the ignorance of CNN to simulate human visual characteristics. Therefore, the entire object detection network is often huge, and the recognition accuracy is not high.

Receptive field (RF) is an important concept that combines biological vision research to reveal why CNN can complete various visual tasks. RF defines the original image's area size that can be seen by a pixel in the different depths feature layers of the CNN [17]. It is precisely the use of the feature that the spatial connection of pixels is local. Just like humans seeing external images through a local receptive field, each neuron does not need to feel the global vision, but only the local image area. Then, at a higher level, these neurons that sense different localities can be synthesized to obtain global information [18]. With the deepening of research on the receptive field, Luo et al. found that not all pixels in the receptive field contribute the same to the output vector. The pixel located at the center of the receptive field contributes the most to the output features. The pixels located at the edges around the receptive field contribute less to the output features; therefore, the RF concept is refined into a theoretical receptive field and an effective receptive field (ERF) [19]. In the object detection task, the CNN output layer's effective receptive field must match the object's size to be detected to accurately and quickly identify and locate the object [20]. Because the ERF size is related to the depth of the network, for the same object detection layer, a large target needs a large ERF, and a small target requires a small ERF. Therefore, it is necessary to consider how to use the ERF and quantify each layer's ERF size to design an effective detection CNN suitable for different scale datasets.

From the above analysis, we understand that studying the visual characteristics of the CNN and adjusting the network depth according to the sample data size are two problems that still need to be solved in the current object detection task. Based on these two problems, we select the anchor-based YOLOv3 in the one-stage object detection method as the research framework, take the electronic components on the PCB as the detection object, take the effective receptive field as the research key point, and finally realize the design of electronic components' detection method based on the anchor size and the effective receptive field size matching. The key contributions of this paper are summarized as follows:

1. We realized the calculation and visualization of the effective receptive field size of different depth layers of CNN based on gradient backpropagation. It not only considers the multichannel problem in the CNN but also regards the processing of nonlinear modules. Through this interpretability analysis, we found that the effective receptive field of different layers changes dramatically. It is easier to understand that the shallow layer is sensitive to position information, and the deep layer is sensitive to semantic information. To the best of our knowledge, this is the first time to reveal how YOLOv3 internally captures the data to detect the target through the receptive field.
2. We proposed a modular YOLOv3 composition strategy. The entire YOLOv3 model is composed of five modules. We can add, remove, and retain some modules. In particular, we found that if we change the number of modules in the backbone network, Darknet-53, the effective receptive field size corresponding to each pixel in the three anchor distribution layers of YOLOv3 will change.
3. We designed an effective receptive field size-anchor size matching algorithm based on YOLOv3. This method analyzes the factors that affect the ERF size of the anchor distribution layer. It formulates module addition and removal strategies to ensure that the ERF size is closest to the anchor size distribution layer of the layer's largest anchor.

In the next section, we review the related works on YOLOv3 and the effective receptive field. In Section 3, we provide an overview of the proposed method. In Section 4, we present experimental results and show the effectiveness of the proposed method. Finally, Section 5 concludes this paper.

## 2. Related Work

Since the proposed PCB electronic components' detection network is implemented on the YOLOv3, which involves the accurate quantification of the ERF size, the following will introduce these aspects' relevant research work.

### 2.1. YOLOv3

The YOLOv3 algorithm is a typical one-stage object detection algorithm that combines the classification and target regression problems with an anchor box, thus achieving high efficiency, flexibility, and generalization performance [21]. Since the YOLOv3 was proposed, it has been used in various object detection tasks [22–24].

When the YOLOv3 performs object detection, the core content has four parts. The first is preprocessing training data, including size cropping of input pictures, generation of clustering anchors, and allocation of anchors. The second part is the feature extraction network, which is mainly completed by DarkNet-53. The third part is the feature fusion network, which uses the YOLO layer to build a feature pyramid. The fourth part is the loss function and the output module. All improvements to the YOLOv3 are around these four parts. The research work in this paper mainly uses the training dataset to resize and generate the anchor, remove or increase the internal module of Darknet-53 to strengthen the matching degree of the anchor size and the effective receptive field size, which is the anchor distribution layer, and finally achieve the goal of efficient detection. So, we mainly reviewed some of the work done by our predecessors in this field.

Liu et al. proposed the ACF-PR-YOLO structure, which includes a region proposal extraction method based on the aggregated channel feature before the whole image was sent to the YOLOv3 for cyclist detection high-resolution pictures [25]. Luo et al. divided the original images into equal parts with k-fold cross-validation, helping them make full use of their train data [26]. Xiong et al. proposed an anchor box and YOLOv3-Darknet model based on adaptive data clustering to identify, classify, and detect dry and wet garbage [27]. Kong et al. proposed a model in Darknet-53 that conducts efficient feature extraction via the Dual-Path Network (DPN) module and the fusion transition module during the real-time sonar target detection [28]. Zhang and Zhu replaced the original Darknet-53 with the Darknet-23 to improve the detection speed when detecting moving vehicles in aerial infrared image sequences [29]. Li et al. employed depthwise separable convolution to design the backbone network to reduce the parameters and the extract crack features effectively for crack inspection in aircraft structures [30]. Ma et al. replaced the Darknet-53 CNN in the YOLOv3 with the lightweight CNN ShuffleNetv2, and the improved YOLOv3 model was effective for the detection of collapsed buildings in post-earthquake high-resolution remote sensing images [31]. Pang et al. replaced the Darknet-53 with the Darknet-13 in the YOLOv3, achieving the object detection task concealed under people's clothing [23]. Wang and Zhang replaced the backbone Darknet-53 in the YOLOv3 with the Darknet-19, and the training speed greatly improved since the residuals' network was not added to the Darknet-19 on scene text detection [32]. Zhang et al. designed a DB-Darknet-53 feature extraction network embedded in the inception structure to solve the problem that features are likely to be lost in the feature extraction process, the YOLOv3, which effectively reduces the loss of features [33]. Won et al. proposed increasing the recognition speed by decreasing the Darknet-53 to 24 layers [34]. Zhang and Zhu introduced the sloping anchor box to overcome the flaws of the traditional horizontal bounding box, which is intended to predict the target position and angle [35].

### 2.2. Effective Receptive Field

Since the RF concept is connected with the CNN, researchers have been trying to use the RF to reveal the internal reasons as to why CNN can perform some visual tasks. In particular, several mathematical formulas are used to describe the relation of the convolution kernel size, convolution padding size, convolution stride size, convolution dilation rate, and the size of different feature layers' receptive fields [36, 37]. Regarding the receptive field, the most widely used in CNN is to build a multi-receptive field module.

The concept of the ERF (effective receptive field) comes from a problem. Since increasing the receptive field can improve recognition accuracy, is it necessary to increase the CNN network depth to maximize recognition accuracy? The answer is no. Some researchers noted that a given feature was not equally impacted by all input pixels within its receptive field region: the input pixels near the center of the receptive field had more “paths” to influence the feature and consequently carried more weight. The theoretical receptive field refers to the region observed in the input space for a neuron in the convolutional neural network. The effective receptive field refers to the set of input neurons that are connected to a higher level neuron, excluding the invalid neurons in the receptive field. Luo Wenjie et al. provided a mathematical formulation and a procedure to measure effective receptive fields, experimentally observing a Gaussian shape on the theoretical receptive field, with the peak at the receptive field center [19]. Since this landmark paper's emergence on effective receptive fields, people have begun to use it to do some visual tasks and achieved favorable results. ZHAO Baojun et al. proposed a novel anchor generation method, which takes the effective receptive field as the standard [38]. Liu et al. discussed the relationship between effective receptive field and semantic segmentation model in detail. They proposed the concept of effective receptive field intensity, which could remove the negative values of the gradient map and normalize the values of the gradient map to [0, 1] [39]. Zhang et al. analyzed the relationship between anchor, theoretical receptive field, and effective receptive field in target detection. They designed anchor scales based on the effective receptive field and a proposed equal proportion interval principle on all the common face detection benchmarks [20]. Gao et al. formulated a theoretical framework for analyzing ERFs, from which they gained insights to motivate their Deformable Kernels (DKs) for object deformation [40].

### 2.3. Knowledge Gaps

Although significant progress has been made in the two fields mentioned above, there are still some gaps that need to be fulfilled from the review.

In terms of the improved design of the Darknet-53 on the YOLOv3, most studies mainly focused on replacing the backbone Darknet-53 with the existing high-efficiency backbone or the Darknet-M, and the M is random. Few people pay attention to the influence of the number of convolution modules in the Darknet-53 on the original picture's visual recognition effect. In this paper, the Darknet-X modular design method is proposed. On the premise of maintaining at least three down-samplings of the original feature extraction network, we offer a method of removing, reducing, or adding some modules to achieve a backbone network design that matches the detection object's size.

In terms of the effective receptive field concept, most of its applications only describe the relationship between the receptive field, the effective receptive field, and the target size in general, and rarely involve its specific calculation method. Only two articles included the calculation and display of effective receptive fields, and one used the gradient backpropagation to solve the size of an effective receptive field. However, only one channel was used, and only the effect of the convolution and linear operation modules on the effective receptive field was considered. The other only showed the dilated convolution parameters' effect of the effective receptive fields and did not quantify the size of each layer's effective receptive fields of one specific CNN network. In this paper, an ERF calculation method based on gradient backpropagation is proposed. This method can quantify each feature layer's effective receptive field size in the YOLOv3 before the training and provide data support to design an accurate object detection network.

## 3. Methodologies

This research mainly implements a rapid and lightweight model design method suitable for small-scale object detection by reducing and removing backbone network modules. The network uses the YOLOv3 as the basic network, uses electronic components on the PCB as the detection objects, and uses each anchor group's maximum width and height after input images resize to 416 × 416 as the threshold. By analyzing the influence of the different module combinations of the Darknet-53 on the effective receptive field size of the anchor distribution layer in the YOLOv3, an anchor and effective receptive field matching PCB detection algorithm based on effective receptive field analysis are designed. For the convenience of the following description, the method proposed in this article is called ERFAM-YOLOv3.

**Figure 1:**
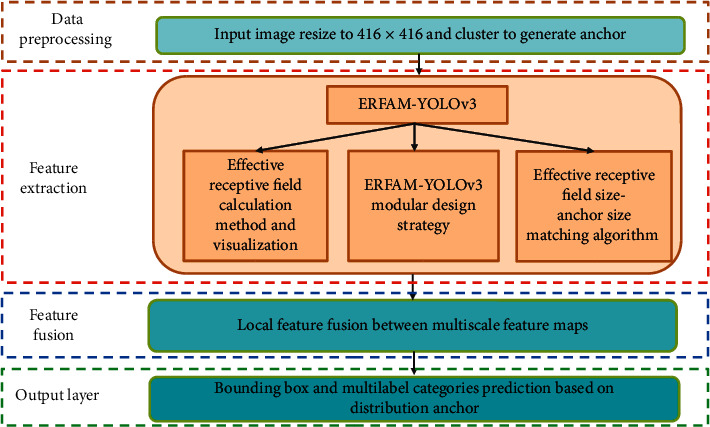
The implementation process of ERFAM-YOLOv3. ERFAM-YOLOv3 is derived from YOLOv3, but the data preprocessing method and feature extraction module are different from the original YOLOv3. The following feature fusion module and output layer are appropriately adjusted according to the changes of the backbone.

The implementation process of the proposed ERFAM-YOLOv3 is shown in Figure 1. It mainly includes four parts, i.e., clustering after resize generates anchor, effective receptive field calculation method, ERFAM-YOLOv3 modular design strategy, and anchor-effective receptive field matching algorithm.

### 3.1. Clustering after Resize Generates Anchor

Anchor box is a concept used by the YOLOv3 when making bounding box prediction. The anchor's significance is that its size predefines the target's most likely height and width to be detected. In the data preprocessing of the YOLOv3, we usually use K-means to cluster the target sizes in the training set to generate nine most likely target anchors, each with its width and height.

**Figure 2:**
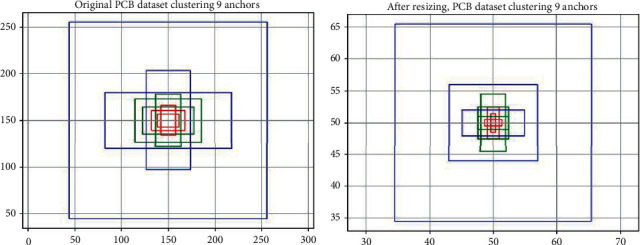
Schematic diagram of K-means cluster anchors. (a) 9 anchors were produced by the original K-means clustering method (b) 9 anchors produced by the K-means clustering method after resized. In this paper, the anchors in (b) are used as a priori box for target recognition and positioning. In YOLOv3, the blue, green, and red anchors will be assigned to the small, middle, and large feature fusion layers of the output layer, respectively.

The size of the pictures in the dataset is often not uniform, and all pictures, whether for training or testing in the YOLOv3, need to be resized to 416 × 416 first. Therefore, in the ERFAM-YOLOv3, the anchors are generated after resizing the training set image in advance. The advantage of this is that all the data are resized in advance to meet the size of the network input, and the width or height of the anchor can be directly used as the threshold of the ERF size in the three anchor distribution layers. After calculation of the PCB train dataset, the traditional sizes of the 9 anchors generated after normalizing are (24 × 14); (16 × 32); (37 × 21); (55 × 29); (28 × 57); (72 × 46); (48 × 106); (136 × 60); and (212 × 211). The sizes of the generated anchor after the picture is resized are (1 × 3); (3 × 1); (2 × 5); (5 × 2); (5 × 5); (4 × 9); (10 × 4); (14 × 12); and (31 × 31). We show them in Figure 2.

### 3.2. Effective Receptive Field Analysis Method

Luo et al. propose the concept of the ERF. He concludes that although all pixels in the receptive field affect the final result, their weights are different. The weight at the center is the largest, and the weight at the edge is the smallest. That means we need to quantify the ERF size to a specific value. This particular value is the original image's effective area size that each pixel in the feature layer of the CNN can see effectively. In the paper [19, 39], the ERF only cares about a critical activation area of a pixel in each feature layer of CNN relative to the original image, regardless of the entire network's weight. We use gradient backpropagation as a more universal and accurate solution method of the ERF size. The complete analysis and solution process is divided into five parts:

- *Load Model*. For any network model that we want to analyze the ERF, we must first load it and put it in the training mode. It is to ensure that we can propagate the gradient back to the original picture smoothly.
- *Set Weights Random*. The CNN can complete many visual tasks because the neural network continuously adjusts the weights in the forward and backpropagation during the learning process. The loss function continues to decline to achieve the training effect. Now we are concerned about which active regions are seen in the input picture when a pixel value is changing in a feature layer of the CNN. Therefore, we set these weights as random values. To avoid the randomness of the calculated ERF size, we finally take the average value of the ERF after 20 random parameter assignments in this paper.
- *Input and Output*. To find the ERF is to take a single pixel on a specific feature layer as the input and use the gradient backpropagation to infer the corresponding activated pixel area under the original model input image size as the output. Therefore, if we want to solve the ERF size of one layer, we use the number of channels of this layer as the number of the input pictures. Each picture's size is determined by the original input picture's width and height forward to the layer. The output is the size of the original image after loading the model with three channels.
- *Tweak Gradients and Backpropagate*. We only want to compute the ERF of one pixel. So we will set the center point of the input corresponding gradient value to 1 and all the others to 0. When we backpropagate this gradient to the output layer, the pixels involved in generating this gradient value will light up, and everything else will be dark.
- *Visualize ERF and Calculate ERF Size*. In the ERF concept proposed by Luo Wenjie, we still use the calculation method that Luo suggested that any pixel with a value greater than 1 (−95.45% of the center point) is considered in the ERF. The ERF size is represented by the square root of the number of pixels within the ERF.

**Figure 3:**
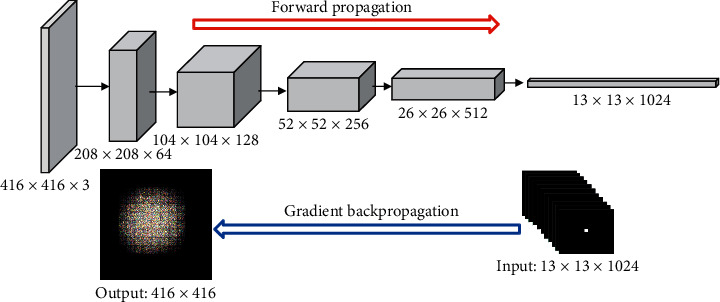
Take the last feature layers of Darknet-53 as input, give random weight to the entire network, and use gradient backpropagation to solve the ERF.

In Figure 3, we take the ERF of the last layer 13 × 13 × 1024 in the Darknet-53 as an example to describe the solution process for the YOLOv3. The calculation method of the ERF size of any other layer is similar.

It is essential to note that the YOLOv3 includes convolution, batch normalization, Leaky ReLU, and multichannel processing. As an activation function, the mathematical expression of Leaky ReLU is

(1) $$\begin{array}{c} \text{Leaky ReLU}\left(x\right)=\left\{\begin{array}{cc} x, & x>0,\\ \alpha x, & x\leq 0,\alpha =0.1. \end{array}\right. \end{array}$$

The gradient backpropagation formula for Leaky ReLU is

(2) $$\begin{array}{c} \frac{\partial L}{\partial x^{l}}=\delta^{l}=\left\{\begin{array}{cc} \delta^{l+1}, & x^{l}>0,\\ \alpha \delta^{l+1}, & x^{l}\leq 0,\alpha =0.1. \end{array}\right. \end{array}$$

Batch normalization is to normalize the *m* outputs of each node of one layer, and the normalized results are outputs. We define values of *x* over a mini-batch *B*={*x*_1_, *x*_2_,…, *x*_*m*_} as input, *μ*_*B*_=(1/*m*)∑_*i*=1_^*m*^*x*_*i*_, *σ*_*B*_^2^=(1/*m*)∑_*i*=1_^*m*^(*x*_*i*_ − *μ*_*B*_)^2^, and $\overset{\wedge }{x_{i}}=\left(\left(x_{i}-\mu_{B}\right)/\sqrt{\sigma_{B}^{2}+\varepsilon }\right)$, and the output is $y_{i}=BN_{\gamma ,\beta }\left(x_{i}\right)=\gamma \overset{\wedge }{x_{i}}+\beta$, where *γ*and *β* are parameters to be learned. The gradient backpropagation formula for batch normalization is

(3) $$\begin{array}{c} \frac{\partial L}{\partial x_{i}}=\frac{\partial L}{\partial \overset{\wedge }{x_{i}}}\cdot \frac{1}{\sqrt{\sigma_{B}^{2}+\varepsilon }}+\frac{\partial L}{\partial \sigma_{B}^{2}}\cdot \frac{2\left(x_{i}-\mu_{B}\right)}{m}+\frac{\partial L}{\partial \mu_{B}}\cdot \frac{1}{m}. \end{array}$$

For the multichannel gradient backpropagation problem, we assume that the loss function *L* is the information of *m* input channels *L*=*g*(*y*_1_, *y*_2_,…, *y*_*m*_), and the output is *n* channels *y*_*i*_=*f*_*i*_(*x*_1_, *x*_2_,…, *x*_*n*_).

The gradient backpropagation formula for the multichannel is

(4) $$\begin{array}{c} \frac{\partial L}{\partial x_{i}}=\sum_{j=1}^{m}\frac{\partial L}{\partial y_{j}}\cdot \frac{\partial y_{j}}{\partial x_{i}}. \end{array}$$

Although the YOLOv3 contains a lot of convolution, BN, Leaky ReLU, and multichannel processing, according to the chain derivation rule and the above effective receptive field analysis method, we can realize the ERF visualization and the ERF size calculation results of any feature layer in the YOLOv3.

### 3.3. ERFAM-YOLOv3 Modular Design Strategy

According to the different arithmetic modules, the modular design strategy of the ERFAM-YOLOv3 is to disassemble the entire YOLOv3 object detection framework. In the reconstruction process, for the different size targets, the original core modules are retained. Some down-sampling modules were removed or added. The number of the repeatable modules is reduced or increased so that the final classification and positioning can be more accurately adapted to the target size.

**Figure 4:**
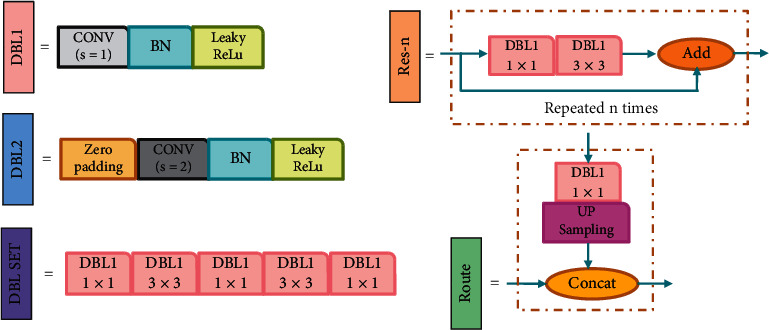
Module composition in the YOLOv3 structure.

According to the operation sequence in the YOLOv3, we have defined five modules, namely, DBL1, DBL2, Res-*n*, DBL SET, and Route.  Figure 4 shows the composition of the five modules. DBL1 is an ordinary convolution module with stride = 1. DBL2 is a down-sampling module with stride = 2. When the picture passes through the DBL2, the output's size will be reduced to half of the input, and the number of channels will be doubled. The Res-*n* module is a residual network that can be repeated *n* times. The DBL SET module prepares for later feature fusion through a series of 3 × 3 and 1 × 1 convolutions. The Route module realizes the fusion of features of the different scales and forms the YOLO layer's output of three levels, namely, large, medium, and small. The three YOLO layers will perform anchor allocation, target classification, and position regression, and finally, achieve object detection. The modular design strategy of the ERFAM-YOLOv3 includes three points. First of all, the combination of feature fusion and the three output layer modules acts as a core module and needs to be retained; secondly, in the Darknet-53, the leftmost DBL1 and the rightmost two down-sampling module groups DBL2 and RES-*n* can be removed or retained; finally, Res-*n* can be repeated *n* times for each occurrence, and *n* is an integer greater than or equal to 0.

**Figure 5:**
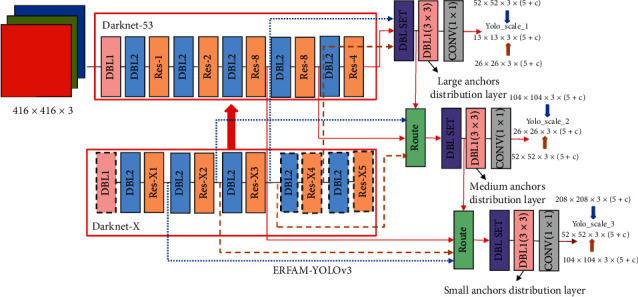
The ERFAM-YOLOv3 structure is designed by a module design strategy.

According to the above modular design strategy, we conducted the modular structure analysis of the original YOLOv3. The ERFAM-YOLOv3 is to retain the core modules, determine which modules need to be removed, and count the number of the repeatable modules to achieve the purpose of matching the three output layers' anchor size to the corresponding layers' ERF size. In this way, the design problem of the ERFAM-YOLOv3 is transformed into the problem that the Darknet-X replaces the original Darknet-53, which is to solve the issues of X1, X2, X3, X4, and X5, respectively, and assess whether some modules need to be removed. The ERFAM-YOLOv3 structure is shown in Figure 5. An ERFAM-YOLOv3 modular design strategy will realize redefinition according to the matching algorithm of anchor size and ERF size, and finally, achieve the object detection function suitable for the target sizes.

### 3.4. Effective Receptive Field Size-Anchor Size Matching Algorithm

From the object detection framework of the YOLOv3, we learned that the object's classification and positioning are achieved by assigning nine predefined anchors to the three output layers of the different scales through continuous learning features of the training data. Although many factors affect the final object detection effect, in the method mentioned in this paper, we are concerned about the size of the ERF corresponding to a pixel of the layer where the anchor is located. We define *d*=ERF size − Anchor size, *d* ≥ 0; the so-called matching is to minimize *d* by adding or reducing repeatability modules and removing some down-sampling modules. The matching degree between the ERF size and the corresponding assigned anchor size is a crucial factor. Because the YOLOv3 has three output ports for object detection, the ideal state is that the ERF sizes of the three anchor distribution layers are, respectively, equal to the maximum width or height of the three anchors allocated in each layer. We can understand from a more vivid explanation that the effective receptive field size and anchor size matching can improve detection. If the ERF size of the corresponding feature layer is much larger than the largest assigned anchor size, then it is like finding a needle in a haystack, and it is easily interfered with by too much context; if the ERF size is far smaller than the smallest assigned anchor size, then the detection is like a blind man touching an elephant, only recognizing local features, not accurately detecting the whole object.

Because the clustering algorithm has obtained the nine anchors' size before training, each anchor's width and height are fixed values. The nine anchors are divided into three groups according to the order from small to large. These three groups are called small anchors, medium anchors, and large anchors in turn. Small anchors are assigned to Yolo-scale-3, medium anchors are assigned to Yolo-scale-2, and large anchors are assigned to Yolo-scale-1. We can see them in Figure 5. We define *a*_max*s*_, *a*_max*m*_, and *a*_max*l*_ as the maximum width and height in each group of anchors. At the same time, we define ERF_1_, ERF_2_, and ERF_3_ as effective receptive field sizes corresponding to large anchors' distribution layer, medium anchors' distribution layer, and small anchors' distribution layer. For the CNN model, the more the modules, the deeper the network and the larger the ERF size corresponding to the output layer. Therefore, under the premise of *X*1 = *X*2 = *X*3 = *X*4 = *X*5 = 1, we take *a*_max*s*_, *a*_max*m*_, and *a*_max*l*_ as the thresholds and set specific discriminant rules. The core of the ERFAM-YOLOv3 lies in minimizing the difference between ERF_1_ and *a*_max*l*_, the difference between ERF_2_ and *a*_max*m*_, and the difference between ERF_3_ and *a*_max*s*_ by removing some modules or increasing the number of repeatable Res-*n*, which is the criterion for anchor and effective receptive field matching.

**Figure 6:**
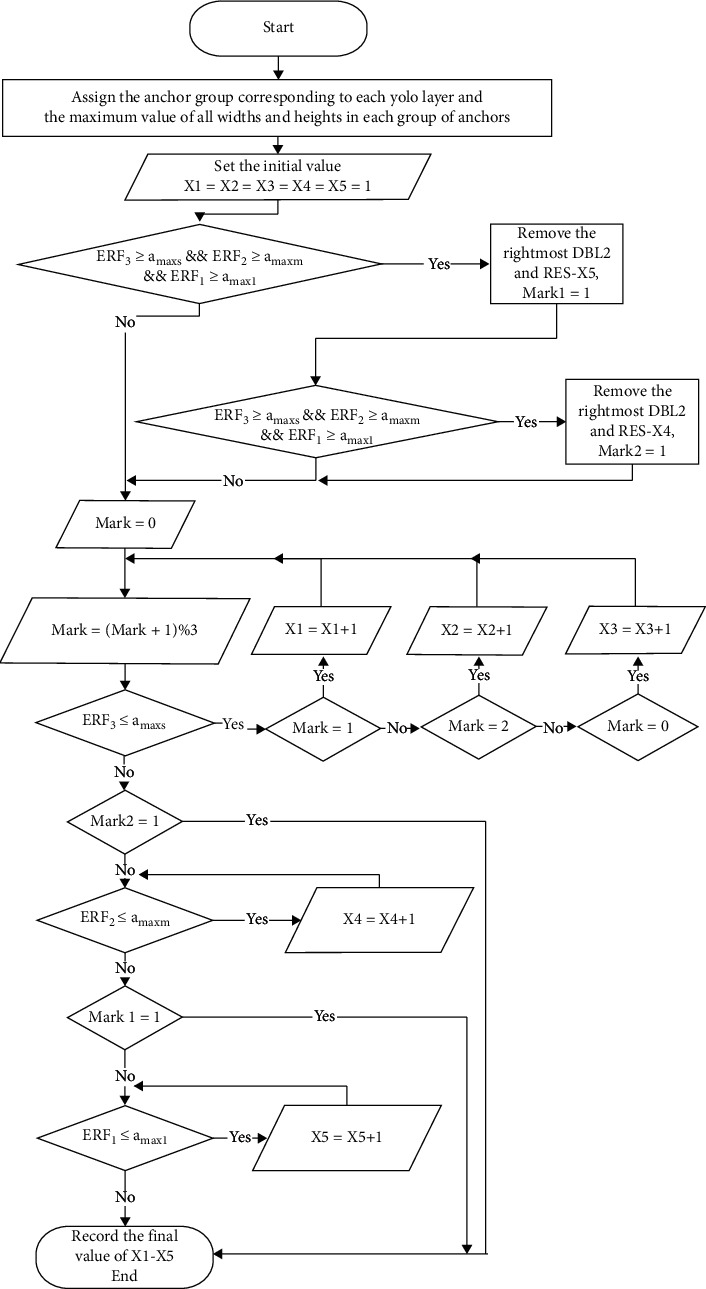
Flow chart of anchor size-effective receptive field size matching algorithm.

We designed the effective receptive field size-anchor size matching algorithm to detect the objects based on the above definition. The flowchart of the effective receptive field size-anchor size matching algorithm is shown in Figure 6. We can learn from the flow chart that there are two cases about matching anchors' size and effective receptive fields' size.

Case one: in the three comparison formulas of ERF_3_ and *a*_max*s*_, ERF_2_ and *a*_max*m*_, and ERF_1_ and *a*_max*l*_, as long as one or more of the former is smaller than the latter, there is no need to remove any module. The three output layers' sizes are 13 × 13, 26 × 26, and 52 × 52, respectively. In this case, we only need to find the values of *X*1 to *X*5 according to the ERFAM-YOLOv3 modular design strategy.

Case two: in the three comparison formulas of ERF_3_ and *a*_max*s*_, ERF_2_ and *a*_max*m*_, and ERF_1_ and *a*_max*l*_, all the former are bigger than the latter. The deeper the network, the larger the effective receptive field corresponding to the anchor distribution layer, and to reduce the *d* between the effective receptive field size and the anchor size, we need to calculate the number of Res-*n* after removing some modules. There are two options for removing modules, and once these modules are removed, they cannot be recovered. Option one: we must delete the rightmost DBL2 and RES-*X*5. Under this option, if (ERF_3_ ≥ *a*_max*s*_)&(ERF_2_ ≥ *a*_max*m*_)&(ERF_1_ ≥ *a*_max*l*_) does not hold, the three output layers' sizes are 26 × 26, 52 × 52, and 104 × 104, respectively. We just need to solve *X*1–*X*4. If the fifth down-sampling DBL2 and Res-*X*5 are deleted, the situation (ERF_3_ ≥ *a*_max*s*_)&(ERF_2_ ≥ *a*_max*m*_)&(ERF_1_ ≥ *a*_max*l*_) still occurs, and option two will appear. Option two: based on deleting the fourth down-sampling DBL2 and Res-*X*4, we continue to delete the fourth down-sampling module and RES-*X*4. The three output size layers are 52 × 52, 104 × 104, and 208 × 208, respectively. Under this option, we only need to calculate the value of *X*1–*X*3 to complete the effective receptive field size-anchor size matching algorithm.

The anchor effective receptive field matching algorithm is to increase, decrease, and remove the number of modules in the Darknet-53 to minimize the difference between the anchor distribution layer's effective receptive field size and the anchor size to achieve the best match to improve the object detection effect.

## 4. Experiments

We evaluate the proposed method on a dataset of PCB electronic components. There are 1000 images, 29 instrument categories, and 182900 electronic components in the dataset [15]. We first analyzed the ERF of each feature layer of the YOLOv3. In particular, we found that the effective receptive fields of the three yolo-scales are much larger than the assigned anchor size. Using the modular design strategy and the anchor effective receptive field matching algorithm, we designed the ERFAM-YOLOv3 suitable for electronic component detection on PCB.

### 4.1. Experimental Platform and Parameter Setting

The experimental platform is the operating system (OS): Windows 10, core processor (CPU): Intel Xeon 6132 × 2 2.60 GHz, graphics processor (GPU): NVIDIA Titan RTX (24 G), hard disk space: 512 G SSD + 2T SATA, memory: 192 GB, Python 3.5.2. program development framework: Python 3.7, TensorFlow 2.0, CUDA 10.1. The PCB electronic component dataset is divided into 8 : 2, that is, eight pieces of data are randomly selected for training, and two pieces of data are used as detection data.

### 4.2. From the YOLOv3 to the ERFAM-YOLOv3

The ERFAM-YOLOv3 design suitable for electronic component detection on the PCB will be completed using the ERF and the effective receptive field size-anchor size matching algorithm.

#### 4.2.1. Analysis and Calculation of the YOLOv3 ERF

To get the ERF size of each feature layer of the YOLOv3, we must first load the YOLOv3 model. Each convolutional layer has two parameters—weight and bias. We will set every layer's weight to be a random value and the bias to be 0. The BN layer has four parameters—weight, bias, running_mean, and running_var. We set the weight to be a random value, bias to 0, running_mean to 0, and running_var to 1.

**Figure 7:**
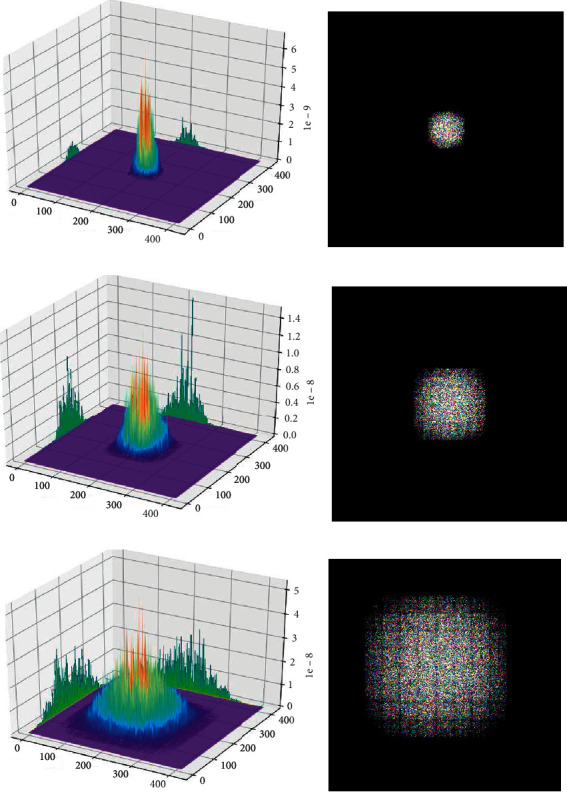
YOLOv3 original anchors allocation corresponding to the activation map and effective receptive field. (a) yolo_scale_3 (52 × 52) activation map (b) yolo_scale_3 (52 × 52) ERF (c) yolo_scale_2 (26 × 26) activation map (d) yolo_scale_2 (26 × 26) ERF (e) yolo_scale_1 (13 × 13) activation map (f) yolo_scale_1 (13 × 13) ERF.

We are particularly concerned about whether the ERF size of the three yolo_scale output layers corresponding to the assigned anchors matches the anchors' size. Therefore, Figure 7 shows the degree of participation of a single-pixel in the three output layers corresponding to pixels in the original image and the calculated ERF size. At the same time, we provide feedback to the original image from a pixel point of the input feature layer, find the point with the strongest activation degree, and find the square root of the number of all pixel points with the activation degree within 95.45% of the strongest point value, to obtain the effective receptive field size of the layer.  Table 1 shows the ERF size of the three yolo_scale layers of the YOLOv3.

#### 4.2.2. ERFAM-YOLOv3: Suitable for Electronic Component Recognition on PCB

As we all know, there are many electronic components on the PCB, and many components are soldered to the PCB through SMT (Surface Mounted Technology). They have similar shapes and small sizes. According to the previous analysis of the electronic component dataset, we can understand from Table 1 the distribution of the three groups of anchors in the three output layers of the YOLOv3 and the maximum width and height of each group. By comparing the corresponding output layer's ERF size, we found that the anchor size of each output layer is far smaller. Since the field of view is too large, it is easy to ignore the target during object detection and the problem of missing the object occurs.

**Figure 8:**
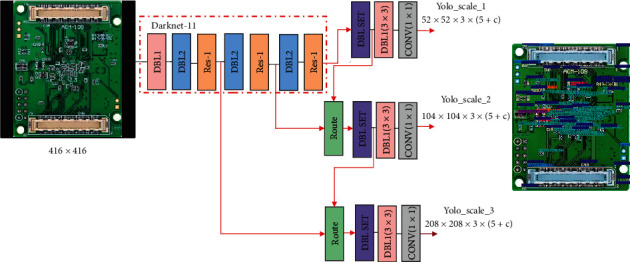
ERFAM-YOLOv3 network structure.

According to the effective receptive field size-anchor size matching algorithm, we need to remove the fifth down-sampling DBL2 and RES-*X*5 in the Darknet-53 and remove the fourth down-sampling DBL2 and Res-*X*4 modules, and also get *X*1 = *X*2 = *X*3 = 1. That is to replace the original Darknet-53 with the Darknet-11. At the same time, the original yolo_scale_1 changed from 13 × 13 to 52 × 52, yolo_scale_2 changed from 26 × 26 to 104 × 104, and yolo_scale_3 changed from 52 × 52 to 208 × 208. The final ERFAM-YOLOv3 network structure is shown in Figure 8.

#### 4.2.3. Analysis and Calculation of the ERFAM-YOLOv3 ERF

**Figure 9:**
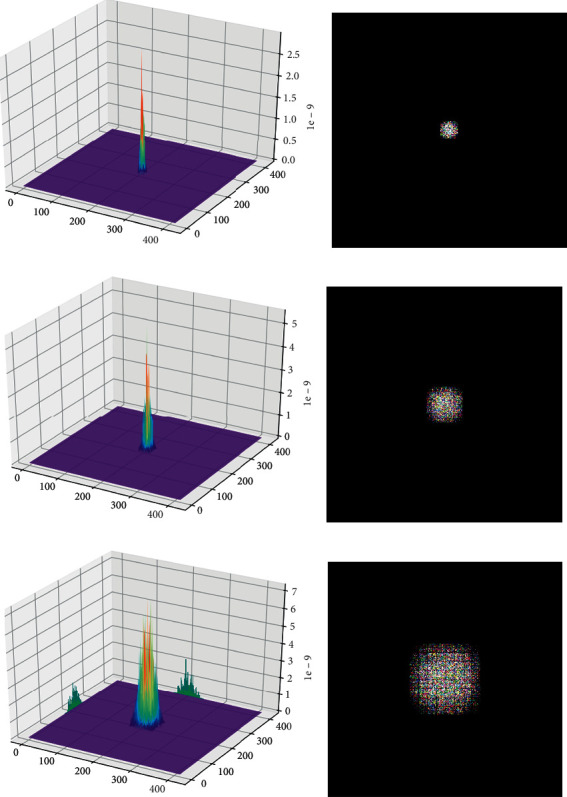
ERFAM-YOLOv3 anchors allocation corresponding to the activation map and effective receptive field. (a) yolo_scale_3 (208 × 208) activation map (b) yolo_scale_3 (208 × 208) ERF (c) yolo_scale_2 (104 × 104) activation map (d) yolo_scale_2 (104 × 104) ERF (e) yolo_scale_1 (52 × 52) activation map (f) yolo_scale_1 (52 × 52) ERF.

After completing the design of the ERFAM-YOLOv3 object detection framework adapted to the electronic component dataset, we again use the previous ERF analysis and calculation methods to perform a single pixel of the three anchor distribution output layers of ERFAM-YOLOv3 corresponding to the original image. The activation map and ERF are shown in Figure 9.

#### 4.2.4. Analysis and Discussion of the ERF about the YOLOv3 and the ERFAM-YOLOv3

Comparing Figure 7 with Figure 9, we can intuitively see that the activation map's peak corresponding to the ERFAM-YOLOv3 for each yolo_scale layer is generally higher than that of the YOLOv3. For example, the peak of the activation map of the yolo_scale_1 in the ERFAM-YOLOv3 is 1.9 × 10^−9^, the peak of the activation map of the yolo_scale_1 in YOLOv3 is only 4.3 × 10^−9^, and the other two layers are similar. The comparison shows that in the YOLOv3 network, the output layer corresponding to each pixel in the original image is more involved in the calculation than the same pixel in the ERFAM-YOLOv3. At the same time, we found that the ERF corresponding to each yolo_scale in the ERFAM-YOLOV3 is much smaller than the YOLOv3. The previous analysis means that the deeper the network, the greater the corresponding pixel's activation degree. By calculating the difference of the yolo_scale's ERF size and the anchor's *a*_max_ in Table 1, we find that the *d*_1_=ERF_1_ − *a*_max*l*_ of the yolo_scale_1 is 143 in the YOLOv3 and is 16 in the ERFAM-YOLOV3, the *d*_2_=ERF_2_ − *a*_max*m*_ of the yolo_scale_2 is 86 in the YOLOv3, and that is 14 in the ERFAM-YOLOv3, and the *d*_3_=ERF_3_ − *a*_max*s*_ of the yolo_scale_3 is 44 in the YOLOv3 and is 8 in the ERFAM-YOLOv3.

We found that the above comparative analysis of the YOLOv3 and the ERFAM-YOLOv3 found that for the dataset with a small overall size distribution after clustering, after removing some modules and reducing repeatable number Res-*n*, it can indeed significantly reduce the size difference between the ERF and the anchor. In the effective receptive field size-anchor size matching algorithm proposed earlier, it was clearly defined that the smaller the difference between the effective receptive field size and the anchor size, the higher the matching degree between them. Therefore, we can conclude that the ERFAM-YOLOv3 can effectively improve the matching degree between the anchor and the effective receptive field, and solve the problem that a pixel of the anchor distribution layer corresponds to a too large field of view in the original image.

### 4.3. The Experimental Results

To better illustrate the proposed algorithm's effectiveness, we will represent by showing the detection image results, the accuracy of the table of detection, and a series of curves of the Faster-RCNN, SSD, YOLOv3, and ERFAM-YOLOv3.

#### 4.3.1. Analysis of Subjective Test Results

**Figure 10:**
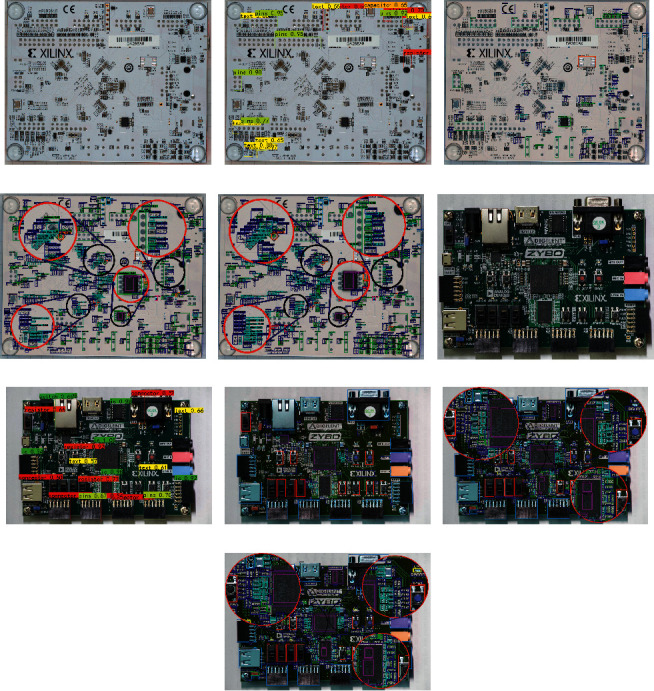
Comparisons of object detection results between the four algorithms. (a) Original image of Arty_Bottom. (b) Faster R-CNN object detection effect of Arty_Bottom. (c) SSD object detection effect of Arty_Bottom. (d) YOLOv3 object detection effect of Arty_Bottom. (e) ERFAM-YOLOv3 object detection effect of Arty_Bottom. (f) Original image of Zybo. (g) Faster R-CNN object detection effect of Zybo. (h) SSD object detection effect of Zybo. (i) YOLOv3 object detection effect of Zybo. (j) ERFAM-YOLOv3 object detection effect of Zybo.

Faster-RCNN, SSD, YOLOv3, and ERFAM-YOLOv3 can realize multi-target detection in one image, and we tested 200 images with them. The identified target is matched in different bounding boxes, and some detection results are expressed in Figure 10. Figures 10(a)–10(e) represent the Arty_Bottom original image and detection effects of the above four algorithm experiments. Figures 10(f)–10(j) represent the Zybo original image and the above four algorithm experiments' detection effects.

In the five pictures about Arty_Bottom in the left column of Figure 10, Figure 10(a) is the original picture. From this picture, we can see that the PCB bottom plate is lighter in color, and there are many targets on the PCB, such as resistors, capacitors, texts, pins, pods, and components waiting for identification.  Figure 10(b) is the recognition effect of Faster-RCNN. In this picture, only a few bounding boxes are drawn. The identified targets' positions are not accurate enough, and a large number of targets are not recognized.  Figure 10(c) is the recognition effect of SSD. From this picture, we can see that the effect of SSD on object detection is better than the Faster-RCNN. The number of identified target boxes is more than the Faster-RCNN, and the box's position can also closely surround the target. But, there are still many targets that cannot be identified, through Figures 10(d) and 10(e) based on the YOLOv3 and the ERFAM-YOLOv3. Most targets can be identified, and the position of the box can accurately surround the target. To further compare the advantages and disadvantages of the YOLOv3 and the ERFAM-YOLOv3, we add a local magnification effect in the figure. We enlarge the small circles at the same position in Figures 10(d) and 10(e) into large circles by comparing the large circles' recognition effects. We can find that the target boxes recognized by the YOLOv3 are relatively sparse for the reason of missing targets, and the ERFAM-YOLOv3 can accurately identify all dense targets.

**Figure 11:**
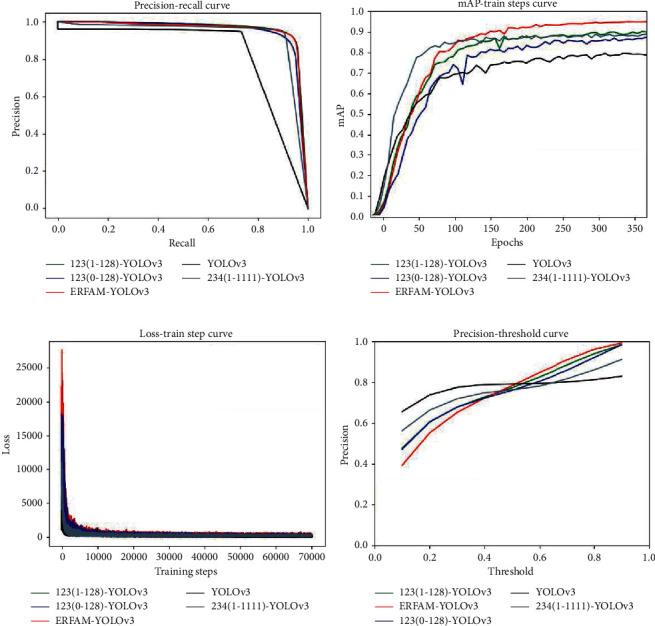
Evaluation curves of the four algorithms. (a) precision-recall curve. (b) mAP-train steps curve. (c) Loss-train step curve. (d) Accuracy-threshold curve.

The right column of Figure 10 shows the different detection effects of Zybo. First of all, Figure 11(a) shows that the PCB bottom plate is darker in color, and the targets on the PCB are large in number and variety, and vary in size. Similar to Arty_Bottom's recognition effect, the Faster-RCNN has the worst detection effect, the number of detection targets is small, and there are detection errors. However, the SSD framework's accuracy is significantly better than the Faster-RCNN; there are still many targets that do not identify it. Both the YOLOv3 and the ERFAM-YOLOv3 have a significant effect on object detection on the PCB. However, it can still be seen from the locally enlarged view that the ERFAM-YOLOv3 is superior to the YOLOv3 in the number of recognized objects and in the accuracy of the position.

#### 4.3.2. Analysis of Objective Test Results

For detecting PCB electronic components containing 29 categories, we used the AP (average precision) of each category of components to characterize the four algorithms' performance. In Table 2, we use bold to indicate that this algorithm's result is better than or equal to other algorithms. From Table 2, we can learn from the specific data that the ERFAM-YOLOv3 has improved detection accuracy in 28 categories.

The comparison of objective analyses shows that the detection effect of the ERFAM-YOLOv3 in Figure 10 is better than the other three detection models. This result is mainly due to the structural improvement of the YOLOv3 neural network, the match of the ERF size and anchor size, and improved detection accuracy on electronic components.

#### 4.3.3. Ablative Analysis

To perform a detailed ablative analysis, we have conducted experiments with the YOLOv3 baseline. We use the mAP (mean average precision) as an evaluation index to measure the model's effectiveness. The higher the value of the mAP, the better the detection effect. For electronic components on the PCB, the mAP of the original YOLOv3 is 79.48%.  Table 3 shows the effectiveness of different components on anchor size and effective receptive field size matching. (1) Remove the fifth down-sampling DBL2, Res-*X*5, and let *X*1 = *X*2 = *X*3 = *X*4 = 1. Because the three outputs of the object detection model at this time come from the second, third, and fourth down-sampling outputs of the original model obtained by feature fusion, the model is named 234(1-1111)-YOLOv3. The first 1 in the brackets means that the leftmost DBL1 of the Darknet-53 is reserved, and the following four numbers mean that the values of *X*1–*X*4 are 1, respectively. 234(1-1111)-YOLOv3 compared with YOLOv3, and mAP increased by 7.17%. (2) Remove the fourth down-sampling DBL2, Res-*X*4, and let *X*1 = 1, *X*2 = 2, *X*3 = 8. Because the three outputs of the model come from the first, second, and third down-sampling modules of the original YOLOv3, we named them 123(1-128)-YOLOv3. 123(1-128)-YOLOv3 compared with 234(1-1111)-YOLOv3 and mAP increased by 3.23%. (3) Based on the above operations, continuing to remove the leftmost DBL1 module not only did not improve mAP but also reduced mAP by 2.70%. This operation shows that DBL1 is useful in object detection tasks and cannot be removed. We call this model 123(0-128)-YOLOv3. The first 0 in the brackets means that we have removed the leftmost DBL1 in the Darknet-53 and the 128 after representing the values of *X*1, *X*2, and *X*3. (4) Finally, based on (1) and 2), we set *X*1 = *X*2 = *X*3 = 1. Because this is the model with the highest matching degree between the anchor size and the ERF size, we named it ERFAM-YOLOv3, and the mAP of the ERFAM-YOLOv3 can be increased by 5.15% compared to 123(1-128)-YOLOv3.

To further illustrate that the different components in Table 3 can enhance the matching degree of the ERF size and the anchor size, we also calculated the results *d* when removing other modules in the Darknet-53, as shown in Table 4.

From Tables 3 and 4, we know that for the small-size electronic component detection network on the PCB, with the removal of the fifth and fourth down-sampling modules, Res-*X*5 and Res-*X*4, the difference between the ERF size and the corresponding anchor size is getting smaller and smaller, that is, the matching degree is getting higher and higher. The detected mAP is also getting higher and higher.

#### 4.3.4. Analysis of a Series of Curves

To comprehensively compare the advantages and disadvantages of different object detection algorithms in the above ablation experiments, we have drawn four curves for comparative analysis in Figure 11. Among the four curves, we use red to represent the ERFAM-YOLOv3, green for the 123(1-128)-YOLOv3, blue for the 123(0-128)-YOLOv3, gray for the 234(1-1111), and black for the YOLOv3.

The precision-recall is a useful measure of the success of prediction when categories are very imbalanced. In information retrieval, precision is a measure of result relevancy, while recall is a measure of how many truly relevant results are returned. With the accuracy of the *y*-axis and the recall rate of the *x*-axis, we get the precision-recall (P-R) curve. The P-R curve shows the tradeoff between precision and recall of different thresholds. The higher the accuracy, the higher the recall rate, and our models and algorithms are more efficient. That is, the drawn P-R curve is as close as possible to the upper right. We can see from Figure 11(a) that the red line is closest to the upper right and encloses the other four YOLOv3 algorithm curves. Therefore, the ERFAM-YOLOv3 algorithm is represented by the color red and shows the best performance.

The mAP provides a single-figure measure of quality across recall levels. Among evaluation measures of different object detection algorithms, mAP has shown to have excellent discrimination and stability. In this paper's experiments, the number of training we set is 350 epochs, which means 70,000 steps. We have the mAP value as the *y*-axis, and the range is from 0 to 100%; the number of iterations is the *x*-axis, which ranges from 0 to 350. We can see from Figure 11(b), although 234(1-1111)-YOLOv3 first reached stability, the mAP of ERFAM-YOLOv3 climbed the highest and finally reached 95.03%, indicating that the ERFAM-YOLOv3 model has the best performance in terms of object detection accuracy.

Figure 11(c) shows the loss value curve changes with iterations of the five algorithms. It can be seen that the five lines correspond to the algorithm quickly fitting in the first 8000 steps, then the loss gets smaller speedily, and then gradually stabilizes after 12,000 steps. The loss value of the ERFAM-YOLOv3 is the highest at the starting point, but it can also reach the same stable value as the other four lines after 15,000 steps.

Figure 11(d) shows the relationship between the precision and confidence of object detection. It is known from Figure 11(d) that as the confidence level increases, the accuracy of object detection also increases. During detection, the precision and confidence provided by the red line model are significantly higher than the other four algorithms, reflecting the superiority of the ERFAM-YOLOv3 algorithm.

#### 4.3.5. Analysis of Algorithm Complexity

We can describe its detection effect with detection accuracy and model modeling power (FIOPs), and model size (parameters) to describe model complexity for a deep learning framework. FLOPs (floating-point operations, *s* tables complex numbers) refer to floating-point operands, understood as the calculation amount. FLOPs represent the required computing power. Parameters describe the needed memory. Usually, they are weights that are learned during training. The more parameters in the network, the more parameters need to be trained, resulting in a longer training time.

We measured the mAP, FIOPs, and parameters of the Faster-RCNN (Resnet-50), SSD (VGG16.512 × 512), YOLOv3 (Darknet-53), and ERFAM-YOLOv3 (Darknet-11) involved in this paper. They are shown in Table 5. From Table 5, we can find that the ERFAM-YOLOv3 has the highest mAP of 95.03%, which is 15.55 points higher than the original YOLOv3, 50.02 points higher than the SSD, and 70.4 points higher than the Faster-RCNN. The parameter amount of the ERFAM-YOLOv3 is also the least one of the four algorithms, especially compared with the original YOLOv3; the parameter amount is only 35.61% of the original parameter amount. From the perspective of FLOPs, the lower the computing power required for the algorithm, the better. We can see although the FLOPs of ERFAM-YOLOv3 is not the lowest, only more than the FLOPs of YOLOv3 is 4.099G, the calculation complexity of ERFAM-YOLOv3 is still very low. In summary, the ERFAM-YOLOv3 has high detection accuracy, a small model, and low computational complexity, making it an ideal object detection framework for electronic components.

### 4.4. Discussion

Use the matching of the ERF size and anchor size as the object detection network design's entry point. The ERFAM-YOLOv3 enjoys significant advantages of high detection accuracy, being lightweight, and with low operation complexity compared with the traditional object detection algorithm. The benefits attributed to considering the internal reason as to why the CNN can perform various visual tasks. The area where a single pixel of the output layer is mapped to the original image is fixed, and only some pixels in this area are effective. The predefined object anchor, far smaller or larger than the ERF size, will bring low efficiency and low accuracy of detection. The ERF analysis method mentioned in the ERFAM-YOLOv3 can not only calculate and visualize the ERF of the three anchor distribution layers but also can be applied to any feature layer of the CNN for the ERF analysis, which, in turn, can form a hierarchical explanatory analysis. From a technological point of view, the modular network design provides packaged and encapsulated modules with independence, addability, removal, and core, which can be constructed by building blocks to complete recognition networks suitable for different size targets. To show that the module recombination based on ERF Sizer-Anchor Size matching can improve the detection effect, we made two comparisons with the electronic components on the PCB as the detection target. The first shows the differences between the ERF size and the anchor size in YOLOv3, ERFAM-YOLOv3, and the other three algorithms were formed after different modules are removed. The *d* reflects their matching. The second shows the difference of the backbone in the YOLOv3 and the ERFAM-YOLOv3, from the Darknet-53 to the Darknet-11. These two changes confirm that the maintenance, removal, and addition/reduction of modules significantly influence the ERF size. The above analysis provides an interpretable basis for us to design detection networks that adapt to different size targets.

Although the ERFAM-YOLOv3 obtains many significances, such as high accuracy, fast operation, and small parameters, there are still several potential limitations and challenges for further improving its effectiveness. Firstly, the ERF is currently the square root within a specific range of the activation area, so it is friendly to approximately square targets. The shape of the target to be detected is complicated. For example, the existence of a large number of narrow and long objects will limit the matching effect of the ERF size and the anchor size. Secondly, the ERF size is determined by the threshold of 95.45% in the activation map according to the normal distribution. How to adaptively select the threshold is still challenging. Fortunately, according to the current experiences, deformable kernel networks [40], selective kernel networks [41], and principal components analysis [42] may solve the above issues. Therefore, future directions related to the ERF will focus on studying the influence of the different forms of convolution kernels and convolution kernels of different connection methods on the ERF to achieve control of the ERF shape, studying the distribution of principal components in the activation map, and finally, be able to find the optimal threshold of ERF.

## 5. Conclusion

Inspired by the receptive field in biological neuroscience, this paper studied the stimulus (assign anchor) of a pixel in the anchor distribution layer of the YOLOv3, and uploaded it to the original image area by the weights that connect the front and back layers of the CNN, and determined the effective area size (ERF) that cause the stimulation. For the first time, matching the bottom-level anchor size and the top-level ERF size are related to the increase and decrease of the CNN modules. We use the ERF size and the anchor size matching YOLOv3 for the electronic component detection task on the PCB in electronic product manufacturing.

Based on the results presented in this paper, several contributions are of significance. Firstly, a novel ERFAM-YOLOv3 architecture for the object detection of electronic components on the PCB is proposed. Its backbone network, the Darknet-11, is 42 fewer convolutional layers compared to the YOLOv3's backbone network, the Darknet-53. Secondly, the modular composition strategy of the YOLOv3 is designed. It provides the possibility of effective receptive field size changes. Thirdly, the effective receptive field size-anchor size matching algorithm is developed. It provides the feasibility of object detection adapted to different distribution sizes.

The evaluation experiment demonstrates that an effective receptive field size and anchor size matching algorithm based on YOLOv3 could achieve higher detection accuracy and lower model complexity than the other state-of-the-art methods.

## Figures and Tables

**Table 1:** Anchors and ERF size of Yolo_scales inYOLOv3 and ERFAM-YOLOv3.

Anchor	The maximum width or height in each group of anchors	YOLOv3 (Darknet-53)		ERFAM-YOLOV3 (Darknet-11)	
		Output layer	ERF size	Output layer	ERF size
(10 × 4)	31	Yolo_scale_1 (13 × 13)	174	Yolo_scale_1 (52 × v52)	47
(14 × 12)					
(31 × 31)					
(5 × 2)	9	Yolo_scale_2 (26 × 26)	95	Yolo_scale_2 (104 × 104)	23
(5 × 5)					
(4 × 9)					
(1 × 3)	5	Yolo_scale_3 (52 × 52)	49	Yolo_scale_3 (208 × 208)	13
(3 × 1)					
(2 × 5)					

**Table 2:** AP for each electronic component category of four algorithms.

Category	AP (average precision) of algorithm			
	Faster R-CNN (%)	SSD (512) (%)	YOLOv3 (Darknet-53) (%)	ERFAM-YOLOv3 (Darknet-11) (%)
Resistor	28.07	15.89	23.89	**91.29**
Capacitor	14.75	16.08	39.87	**93.91**
Text	14.90	16.72	51.74	**95.15**
Unknown	22.84	43.06	72.62	**97.41**
Emi	17.63	36.36	93.84	**100.00**
Ferrite	23.81	37.24	60.89	**97.39**
Pads	21.09	16.92	48.79	**89.06**
Led	13.51	17.18	68.95	**99.36**
Zener	21.80	75.00	**100.00**	78.79
Component	16.99	17.02	47.05	**72.97**
Transistor	25.21	18.00	93.49	**99.94**
Diode	26.62	27.11	78.76	**100.00**
Jumper	23.26	27.27	80.55	**99.73**
Inductor	23.54	27.27	77.76	**90.33**
Fuse	25.73	27.27	**100.00**	**100.00**
Electrolytic	25.19	27.05	63.01	**89.25**
Transformer	28.24	97.73	**100.00**	**100.00**
Potentiometer	23.45	9.09	55.88	**70.59**
Pins	19.14	54.55	99.00	**98.90**
Clock	16.76	45.45	84.31	**96.08**
Battery	29.85	54.55	**100.00**	**100.00**
Button	27.43	90.43	99.08	**100.00**
Ic	20.02	54.55	95.12	**97.52**
Switch	24.73	81.82	**100.00**	**100.00**
Test	55.02	17.21	74.54	**98.88**
Connector port	31.00	54.52	95.73	**99.43**
Buzzer	34.69	100.00	**100.00**	**100.00**
Heatsink	32.35	100.00	**100.00**	**100.00**
Display	26.64	100.00	**100.00**	**100.00**

**Table 3:** The effectiveness of different components on mAP.

Remove the fifth down-sampling DBL2 and Res-X5, *X*1 = *X*2 = *X*3 = *X*4 = 1	Remove the fourth DBL2, Res-X4, *X*1 = 1 X2 = 2 X3 = 8	Remove the leftmost DBL1, *X*1 = 1 X2 = 2 X3 = 8	*X*1 = *X*2 = *X*3 = 1	mAP@0.5
√				86.65%^+7.17^
√	√			89.88%^+3.23^
√	√	√		87.18%^−2.70^
√	√		√	95.03%^+5.15^

**Table 4:** The matching degree of removing different modules.

Difference between ERF size and anchor size	YOLOv3	234(1-1111)-YOLOv3	123(1-128)-YOLOv3	ERFAM-YOLOv3
*d*_1_	143	63	18	**16**
*d*_2_	86	43	17	**14**
*d*_3_	44	19	10	**8**

**Table 5:** Statistics of accuracy and complexity of four algorithms.

Model	mAP (%)	Params (M)	FLOPs (G)
Faster R-CNN (Resnet50)	24.63	43.435	742.473
SSD (VGG16, 512)	45.01	28.516	91.545
YOLOv3 (Darknet-53)	79.48	61.727	**65.685**
ERFAM-YOLOv3 (Darknet-11)	**95.03**	**21.98**	69.784

## Data Availability

The data used to support the findings of this study are available from the corresponding author upon request.
